# Influenza or Meningococcal Immunization During Pregnancy and Mortality in Women and Infants: A Pooled Analysis of Randomized Controlled Trials

**DOI:** 10.1097/INF.0000000000002629

**Published:** 2020-04-16

**Authors:** Dayna R. Clark, Saad B. Omer, Milagritos D. Tapia, Marta C. Nunes, Clare L. Cutland, James M. Tielsch, Niteen Wairagkar, Shabir A. Madhi

**Affiliations:** From the * Department of Epidemiology, Emory University Rollins School of Public Health, Atlanta, GA; † Yale Institute for Global Health; ‡ Department of Medicine, School of Medicine; § Department of Epidemiology of Microbial Diseases, School of Public Health, Yale University, New Haven, CT; ¶ Centre pour le Développement des Vaccins, Bamako, Mali; ‖ Center for Vaccine Development, University of Maryland School of Medicine, Baltimore, MD; ** Medical Research Council: Respiratory and Meningeal Pathogens Research Unit, Faculty of Health Sciences, University of the Witwatersrand, Johannesburg, South Africa; †† Department of Science and Technology/National Research Foundation, Vaccine-Preventable Diseases Research Chair, Faculty of Health Sciences, University of the Witwatersrand, Johannesburg, South Africa; ‡‡ Department of Global Health, Milken Institute School of Public Health, George Washington University, Washington, Columbia; §§ Bill & Melinda Gates Foundation, Seattle, WA; ¶¶ Vaccines For All, Pune, India; ‖‖ The BMGF Supported Maternal Influenza Immunization Trials Investigators Group are listed in the Appendix.

**Keywords:** maternal immunization, influenza, meningococcal, vaccine, randomized controlled trial

## Abstract

This analysis includes pooled data from 2 placebo-controlled maternal influenza immunization trials, with a separate analysis on a meningococcal conjugate vaccine-controlled maternal influenza immunization trial. Maternal influenza immunization was not associated with infant or maternal all-cause mortality in placebo-controlled trials. In the meningococcal conjugate vaccine-controlled trial, there were fewer deaths during low or any influenza circulation weeks among infants whose mothers received meningococcal conjugate vaccine. ClinicalTrials.gov identifiers: NCT01430689, NCT01034254 and NCT02465190.

Influenza is an important cause of morbidity and mortality in young infants.^1^ Pregnant women are also at increased risk of developing severe illness compared with non-pregnant women.^2^ Sanofi’s Vaxigrip has recently received label indication for active immunization in pregnant women, in addition to passive protection of infants less than 6 months of age as maternal influenza immunization can protect infants through the transfer of maternal antibodies.^3^ Multiple randomized controlled trials have demonstrated that maternal influenza immunization is effective in protecting against influenza infection in both mothers and young infants.^4–7^

Meningococcal infection also causes high mortality in infants, who are at high risk of invasive infection.^8^ Vaccination has proven to be a successful strategy for reducing overall meningococcal incidence.^9^ However, meningococcal vaccines are usually administered to infants between 2 and 4 months of age, indicating that prior to vaccination, the only source of protection is maternal antibodies transferred via the placenta.

Exploring the impact of maternal influenza immunization on all-cause mortality in women and infants is important for determining the safety of this intervention. Previous trial reports did not describe a difference in maternal or infant mortality amongst study arms.^5–7^ By pooling data from recent placebo-controlled clinical trials in Nepal and South Africa, power is higher to detect differences in maternal and infant mortality, as well as mortality by periods of influenza circulation. As meningococcal conjugate vaccine (MCV) may have a biologic impact on all-cause mortality, we also separately compared mortality in women receiving influenza immunization during pregnancy and their infants to those who received MCV using maternal influenza immunization trial data from Mali, where MCV was used as a control.

## MATERIALS AND METHODS

Methods, procedures and initial results from the 3 clinical trials have been previously described.^5–7^ Each trial was initially designed as a separate study funded by the Bill & Melinda Gates Foundation, although pooled analyses of selected study outcomes were planned prior to trial completion. Study protocols and procedures were then coordinated between investigators for the comparison of future results, and planned analyses have been outlined in previous publications.^10^ For this analysis, data from Nepal and South Africa trials, which used placebo controls, are pooled. Mali trial data, where MCV control was used, are analyzed separately, as this intervention may have a biologic impact on all-cause mortality and would be inappropriate to pool with placebo-controlled trials.

Each trial included active, proactive surveillance for influenza. Therefore, conventional cutoffs for passive surveillance are not applicable. Study weeks with at least one positive influenza sample collected from women or infants were considered weeks with active influenza circulation. Active influenza circulation weeks were stratified by weeks of high circulation (≥ 0.25% of subjects at risk tested positive for influenza in a week), and low circulation (0% to 0.25% of subjects at risk tested positive for influenza in a week). The cutoffs were chosen based on influenza circulation data from each trial in consultation with teams from each study site to ensure sufficient high and low weeks of influenza circulation, as well as to create a uniform definition to be used for analysis. Data from previous influenza seasons was not used, as there was not consistent data available across study sites.

Poisson regression models were used to estimate incidence rate ratios (IRR). Follow-up time for infants was 6 months in Nepal and Mali and 24 weeks in South Africa. Pooled estimates of all-cause mortality, based on random intercept models, were adjusted for the effects of site. Interaction by site was considered for pooled estimates, and interaction was significant for infant mortality during no influenza circulation weeks. Statistical analyses were performed using Stata version 14.2 (Stata Corp., College Station, TX). The study protocols were reviewed and approved by institutional review boards from the partner entities involved in this analysis.^5–7^ The 3 trials were registered with ClinicalTrials.gov (trial numbers: NCT01430689, NCT01034254 and NCT02465190).

## RESULTS

The pooled analysis included 5809 women (2909 received IIV vaccine and 2900 received placebo control), and 5695 total live eligible infants born (2846 live-births to mothers who received IIV and 2849 live-births to mothers who received placebo control). In Mali, 4193 women were included (2108 received IIV and 2085 received MCV), along with 4105 live-born infants (2064 live-births to mothers who received IIV and 2041 to mothers who received MCV) (Figure S1, Supplemental Digital Content 1, http://links.lww.com/INF/D947). Stillbirths, miscarriages and abortions were excluded. The distributions of maternal characteristics have been shared in previous publications, demonstrating that the intervention and control groups in the individual trials were similar in terms of maternal age, and gestational age at enrollment.^5–7^

In infants, there was no association in mortality between the IIV arm and the placebo arm [IRR: 1.05; 95% confidence interval (CI): 0.76–1.44]. The same was true for mortality in the women (IRR: 0.80; 95% CI: 0.21–2.96) (Table 1). There was similarly no association in pooled placebo-controlled mortality in infants and women during periods of high, low, any, and no weekly influenza circulation (Table 2; Table S1, Supplemental Digital Content 2, http://links.lww.com/INF/D795). In South Africa, there were fewer deaths among infants whose mother had received IIV during weeks with no influenza circulation (Table 2).

**TABLE 1.**
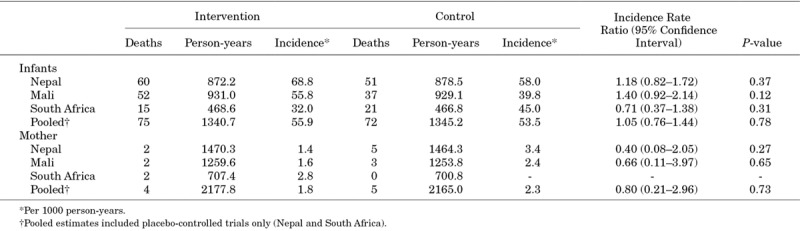
Incidence of Infant and Mother Deaths

**TABLE 2.**
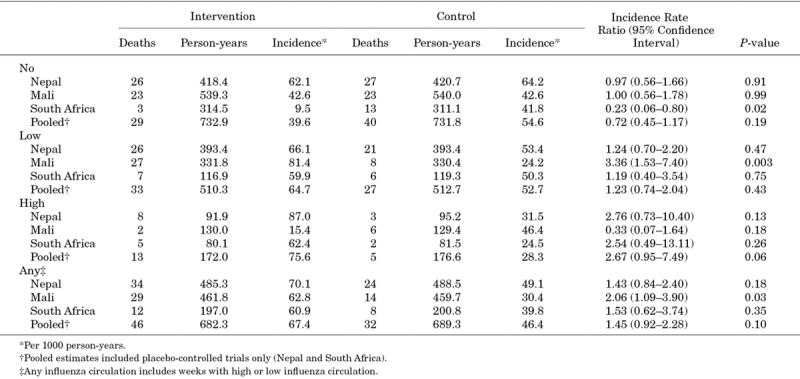
Incidence of Infant Deaths by Weekly Influenza Circulation

In Mali, there was no difference between study arms in terms of infant mortality overall (IRR: 1.40; 95% CI: 0.92–2.14) (Table 1). This was also true during weeks of no and high influenza circulation. However, there was lower mortality in infants in the MCV arm during weeks with low influenza circulation compared with those in the IIV arm (IRR: 3.29; 95% CI: 1.50–7.25). Similar results were obtained when weeks with any influenza circulation were analyzed (IRR: 2.05; 95% CI: 1.08–3.88) (Table 2). The study arms were similar in terms of maternal mortality in Mali, including during weeks of no, low, high or any influenza circulation (Table 1; Table S1, Supplemental Digital Content 1, http://links.lww.com/INF/D795).

## DISCUSSION

Overall, we did not find an association between maternal influenza immunization and infant or maternal mortality in trials using placebo control. In South Africa, however, we observed fewer deaths during weeks with no influenza circulation among infants whose mother had received IIV. Previous pooled analyses of these trials demonstrated that in South Africa, there was also a decrease in severe pneumonia during periods with no influenza circulation, an effect that was not seen in Nepal or Mali.^11^ Therefore, prevention of the sequelae of influenza may lead to protection that lags the influenza season.

Influenza immunization has been reported to decrease mortality in pediatric and adult populations.^12^ However, our findings are consistent with observational studies on maternal influenza immunization in larger populations, which have also not shown a difference in infant mortality between infants whose mother received influenza vaccine and those whose mother did not.^13,14^ There is some evidence that influenza during pregnancy may not be associated with maternal mortality, and it is, therefore, possible that maternal influenza immunization may not be associated with maternal mortality even with a larger sample size.^2,15^ Additionally, pooled analyses of these trials demonstrated safety in terms of negative birth outcomes, including stillbirth (manuscript in press).

Mortality is a statistically rare outcome. Despite combining data from placebo-controlled trials, power in this analysis is likely still too low to detect important differences in mortality between treatment groups. There are also differing point estimates for infant mortality between Nepal and South Africa despite having similar baseline rates of infant mortality, demonstrating that results from a single geography should be interpreted with caution. Despite these limitations, there are few randomized controlled trials for which this data is available, and it is, therefore, important to disclose these findings. Given that these results, along with other available evidence, support current immunization policies in terms of safety, further trials may not be warranted or even ethical.

**FIGURE 1.**
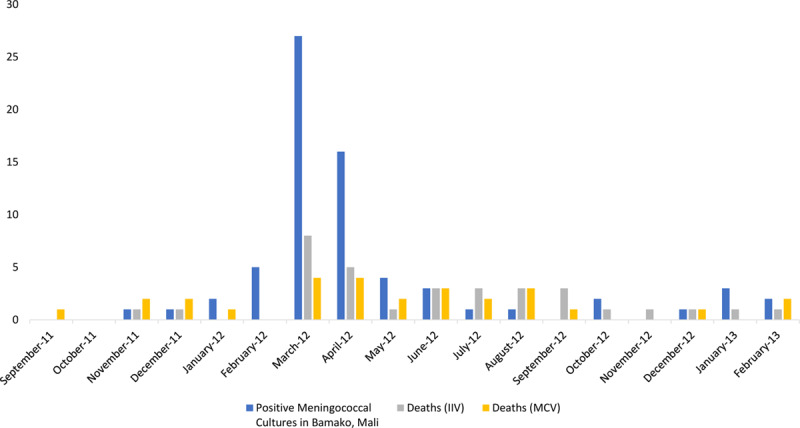
Positive Meningococcal Cultures in Bamako, Mali, and Mali Monthly Deaths by Study Arm.

In Mali, the mortality rate was lower among infants whose mother received MCV during pregnancy compared with those born to women who received IIV during weeks of low or any influenza circulation. As we did not find a difference in infant mortality when placebo control was used, and there was lower incidence of infant mortality in the MCV arm in Mali compared with the placebo arms in Nepal and South Africa, it is unlikely that maternal IIV increased mortality. It may be possible that this estimate was instead driven by a beneficial effect of maternal MCV. In 2002, bacteriology surveillance for invasive bacterial disease in children under 16 years of age began at Hôpital Gabriel Toure (HGT) in Bamako, Mali.^16^ Monthly meningococcal infection data were available from September 2011 to February 2013. When comparing monthly infant deaths in the Mali trial to monthly positive meningococcal cultures collected at the hospital, there tended to be more divergence in the infant deaths between study arms in months with more positive meningococcal cases; there were fewer deaths in the MCV arm compared with the IIV arm (Fig. 1).

Additionally, there is evidence that increased influenza circulation is associated with higher incidence of meningococcal disease during the same week, which may explain why there were few deaths in the MCV arm in weeks with low or any influenza circulation.^17^ A recent analysis of this trial has shown that maternal MCV was successful in transferring protective antibodies to infants.^18^

Although this suggests that maternal MCV vaccine may have been protective against infant mortality, there were no deaths that were known to occur due to meningococcal disease in either study arm in Mali.^6^ It is unlikely that there were high levels of undetected disease due to the design of the study. Severely ill patients were referred to HGT during weekly household visits, where suspected invasive bacterial infections resulted in blood culture, as well as culture of any other normally sterile body fluid. Nevertheless, there was no standard case definition for invasive meningococcal disease among study participants, and cultures of blood or cerebrospinal fluid for those not meeting HGT surveillance criteria were not standard practice. The benefits maternal MCV immunization might have for young infants may be an area for future investigation.

## ACKNOWLEDGMENTS

We would like to acknowledge the late Dr. Mark C. Steinhoff, whose dedication made this work possible. The 3 trials in Mali, South Africa and Nepal as well as this pooled analysis were funded by the Bill & Melinda Gates Foundation. This pooled analysis received research funding from the Bill & Melinda Gates Foundation. Dr. Omer and Ms. Clark serve as consultants to the Bill & Melinda Gates Foundation and received compensation for these services. The terms of this arrangement have been reviewed and approved by Emory University in accordance with its conflict of interest policies.

## APPENDIX

**BMGF Supported Maternal Influenza Immunization Trials Investigators Group:**

Mali: William Blackwelder, PhD; Joseph Bresee, MD; Flanon Coulibaly, MD; Boubacar Diallo; Fatoumata Diallo, MD; Wilbur Chen, MD, MS; Moussa Doumbia, MD; Fadima Cheick Haidara, MD; Adama Mamby Keita, MD, Alexander Klimov, PhD; Mamoudou Kodio, PharmD; Karen Kotloff, MD; Myron M. Levine, MD, DTPH; Vladimir Mishcherkin MS; Uma Onwuchekwa; Sandra Panchalingam, PhD; Marcela Pasetti, PhD; Doh Sanogo, MD; Samba Sow, MD MS; Milagritos Tapia, MD; Boubou Tamboura, PharmD; Ibrahim Teguete, MD; Sharon Tennant, PhD, Awa Traore, PharmD; and John Treanor, MD.

Nepal: Janet A. Englund, MD; Joanne Katz, ScD; Subarna K. Khatry, MD; Jane Kuypers, PhD; Steven C. LeClerq, MPH; Luke C. Mullany, PhD; Laxman Shrestha, MD; Mark C. Steinhoff, MD; and James M. Tielsch, PhD.

South Africa: Peter V. Adrian, PhD; Clare L. Cutland, MD; Andrea Hugo, MD; Stephanie Jones, MD; Locadiah Kuwanda, MSc; Keith P. Klugman, MD, PhD; Shabir A. Madhi, MD, PhD; Kathleen M. Neuzil, MD; Nadia van Niekerk, BTech; Marta C. Nunes, PhD; Justin R. Ortiz, MD; Eric A.F. Simões, MD; Florette Treurnicht, PhD; Marietjie Venter, PhD; Avy Violari, MD; and Adriana Weinberg, MD.
